# Acceptance of a third COVID-19 vaccine dose, vaccine interchangeability, and clinical trial enrolment among parents of children 12–17 years in Lima, Perú

**DOI:** 10.3389/fpubh.2024.1421746

**Published:** 2024-08-14

**Authors:** Carlos R. Celis, Lucie Ecker, Giancarlo Alvarado-Gamarra, Katherine Alcalá-Marcos, Noé Atamari-Anahui, Maria Pia Balmaceda, Kevin Florian, Rodrigo Paredes de la Fuente, Leigh M. Howard, Carlos G. Grijalva, Claudio F. Lanata

**Affiliations:** ^1^Instituto de Investigación Nutricional, Lima, Peru; ^2^Instituto Nacional Cardiovascular “Carlos Alberto Peschiera Carrillo” – INCOR, Lima, Peru; ^3^Unidad de Investigación para la Generación y Síntesis de Evidencias en Salud, Vicerrectorado de Investigación, Universidad San Ignacio de Loyola, Lima, Peru; ^4^Nora Eccles Harrison Cardiovascular Research and Training Institute, University of Utah, Salt Lake City, UT, United States; ^5^Department of Medicine, Mount Sinai West/Morningside Hospital, Ichan School of Medicine at Mount Sinai, New York, NY, United States; ^6^Department of Pediatrics, School of Medicine, Vanderbilt University, Nashville, TN, United States; ^7^Department of Health Policy, Vanderbilt University Medical Center, Nashville, TN, United States

**Keywords:** vaccination, patient acceptance of health care, pediatrics, COVID-19 vaccines, parents

## Abstract

**Objectives:**

To characterize factors associated with parental willingness for their children participation in a COVID-19 vaccine trial, use of different COVID-19 vaccines and acceptance of a third vaccine dose.

**Methods:**

Parents of children aged 12–17 years in Lima, Perú were asked to complete an online questionnaire via social networks, from November 9, 2021, to April 23, 2022. We calculated crude and adjusted prevalence ratios with 95% confidence intervals to compare factors with the mentioned outcomes.

**Results:**

From 523 parents responding, 374 completed the survey. 90.4% would give their children a third vaccine dose, 36.6% would allow their children participation in a COVID-19 vaccine clinical trial, and 33.2% would accept different vaccine brands between doses. Parental belief that COVID-19 vaccine studies met quality standards was associated with acceptance of a third booster dose (adjusted PR 3.25; 95% CI1.57–6.74; *p* = 0.002), enrolment in a COVID-19 clinical trial (adjusted PR 4.49; 95% CI1.25–16.06; *p* = 0.02), and acceptance of different COVID-19 vaccine brands between doses (adjusted PR 10.02; 95% CI1.40–71.95; *p* = 0.02).

**Conclusion:**

Most parents would accept a third vaccine booster dose, approximately a third would participate in COVID-19 vaccine trials. Believing COVID-19 vaccines studies fulfilled quality standards was associated with the study outcomes. It is necessary to inform about the rigorous processes for the development of COVID-19 vaccines to generate confidence in parents to accept these vaccine-related outcomes.

## Introduction

It has been estimated that worldwide more than seventy million children were infected and tens of thousands died with COVID-19 (1). Most cases in children were not severe, but the potential for long-term complications remains unknown to date, particularly in the unvaccinated population. In Perú, thousands of severe cases and deaths were reported in children and adolescents during the Lambda and Omicron wave (2). COVID-19 vaccines for children aged 12 and above were authorized by FDA in May 2021 (3) and became available in Peru by November 2021 (4). A third COVID-19 vaccine dose for adolescents was recommended in Perú in March 2022 (5). Despite this recommendation, booster vaccination intention among this new group of children aged 12–17 years remained low when the vaccination campaign began, motivating us to study the contributing factors.

Vaccine hesitancy for the primary and booster doses in adults has been a significant obstacle to increase vaccination coverage (6). And in parents even more: a systematic review estimated that worldwide, the pooled prevalence of parental acceptance of COVID-19 vaccine for their children was only 57% (7). In the U.S, only half of the adolescents who completed the primary vaccination series received a third dose (8). In contrast, Latin American countries showed less hesitancy and much higher intention to vaccinate with the primary series in adults (9). In Perú, parents had more intention to vaccinate their children (10). However, according to Perú Ministry of Health, approximately 90% of adolescents hospitalized, admitted to intensive care units (ICU) or who died, were unvaccinated (11). Also, even though most adolescents have received at least one COVID-19 vaccine dose, only half of them had received a third dose (12). Determinants for parental hesitancy are related to epidemiological and socioeconomic factors such as education level family income, age and gender; and doubts about vaccine safety (13, 14). In addition, current research shows that literacy in health topics regarding COVID-19 is poor among health care workers and even lower in general population (15).

The World Health Organization and other similar groups are expecting a similar pandemic to COVID-19 in the near future. Therefore, lessons learned about the determinants of parental acceptance of vaccine-related outcomes, based on the experience of the COVID-19 pandemia, will be important. Therefore, we aimed to determine the factors affecting parental willingness to accept a third booster COVID-19 vaccine dose in Lima, Perú for their children aged 12–17 years. Because of the likelihood of receiving more than one brand of COVID-19 vaccines between doses, we also inquired about it, as well as their acceptability for their children to participate in a potential COVID-19 vaccine trial.

## Materials and methods

### Study design and selection of participants

We conducted a cross-sectional study from November 9, 2021, to April 23, 2022, inviting parents of one or more adolescents 12–17 years that resided in Metropolitan Lima, Perú to participate. This period included the time when the Delta variant, characterized by high mortality, was ending (second wave, nov-dec 2021) and when the Omicron variant, characterized by high disease incidence, became predominant (third wave, jan-apr 2022) (16). We used a paid advertising feature on social media platforms (Facebook, Twitter, and Instagram) to diffuse a Google Forms link that included general information about the study, asked for online consent to participate, and then presented the study questionnaire only to residents of Metropolitan Lima with no age restriction for parents. All parents who responded to the survey and met the inclusion criteria were included. We described the design and results of this survey following the checklist for reporting results of internet e-surveys (CHERRIES) (17) to ensure comprehensive reporting.

### The questionnaire and study variables

We developed an online questionnaire using “Google forms” containing 36 questions in Spanish, with focus on three study outcomes: willingness to apply a third boosting vaccine dose, potential participation of adolescents aging 12–17 years in clinical trials, and acceptance to receive different brands of COVID-19 vaccines between the first and second dose (interchangeability).

It also contained multiple-option questions about parent’s characteristics, like epidemiological and sociodemographic factors, general knowledge about COVID-19 vaccines, knowledge about COVID-19 prevention, and parental attitudes on COVID-19 vaccinations [using Likert-scale questions (18)]. The detailed survey can be found in Supplementary material 1.

To develop the questionnaire, we requested the judgements of five specialists in the areas of epidemiology and pediatrics. A summary of the protocol was presented, and they were invited to respond to 10 questions that evaluated the content of the questionnaire, time spent filling it, comprehensibility and coherence with the study objectives. The experts did not have any comment or suggestion about the initial questionnaire. A pilot survey was then applied to the first 50 respondent parents. We verified their understanding and the time consumed by asking for their feedback after every question. Consequently, we decided to reduce the number of questions from 40 to 36 because of redundance and reformulate the phrasing of some questions that were difficult to comprehend. We did not include these answers in the final data analysis.

The estimated time for completion of the final questionnaire was 20 min. Parental participation was voluntary, and no rewards or incentives were used by the research team. Participants had the opportunity to change their responses during the questionnaire, but not once they submitted their final answers. If a subject ended the questionnaire before submitting it, no data was recorded. All responses were anonymous and no personal information was linked to the survey data. Access to the database was limited to the investigators and members of the research team to ensure confidentiality.

### Statistical analysis

Data analysis was performed using STATA v.16 software (StataCorp LP, College Station, Texas, United States). Results of the descriptive analysis for numerical variables were reported using means with standard deviation (SD) or median with interquartile range (IQR); categorical variables were reported with absolute and relative frequencies. Chi-square test or Fisher’s exact test were used for categorical variables. For the exploratory bivariate analysis, Student’s T-test or Mann–Whitney U test were used to compare continuous variables, *p*-values <0.05 were considered as statistically significant. Assumptions for each statistical test we used were checked and validated. To determine the distribution of the data, we applied the skewness and kurtosis criteria as well as the histogram method. To explore the strength of the association, we estimated crude and adjusted prevalence ratios (PR) and 95% Confidence Intervals (CI) using GLM family Poisson with robust variance. To develop the multivariate model, we chose the factors that presented a *p*-value <0.05 in the crude analysis.

### Ethical approval

The study was performed in compliance with relevant laws and guidelines. It was approved by the institutional review board of the Instituto de Investigación Nutricional (411-2021/CIEI-IIN). Upon accessing the survey through the social media advertised link, participants were directed to an introductory page where comprehensive information about the study objectives, procedures, risks and benefits were explained. This page also contained the contact information of the principal investigator in case they had any question. If subjects agreed to participate, they were required to select “yes” on a checkbox before they had access to the full online questionnaire.

## Results

After posting the invitation, from 523 parents responding, 374 (71.5%) met the inclusion criteria (Supplementary material 2). 60% of the parents were recruited between January and April 2022 (third wave of COVID-19, Omicron predominance). Despite 97% (363/374) of the responding parents had already received a COVID-19 vaccine, only 55% of their children had been vaccinated. Other characteristics of participating parents and their children are described in Table 1.

**Table 1:** Characteristics of participating parents with children aged 12–17 years on an on-line survey, in Lima-Perú Nov 2021 – Apr 2022.

Characteristics	*n* (%) (*n* = 374)
Participants, according to the predominant variant by epidemiological wave	
Delta variant predominance (November and December 2021).	149 (40)
Omicron variant predominant (January and April 2022).	225 (60)
Place of residence	
Peri-urban Lima^a^	219 (59)
Urban Lima^b^	155 (41)
Parents’ age (years)^c^	42.8 ± 7.4
Relationship to children	
Father	57 (15.2)
Mother	317 (84.8)
Parental level of education	
Less than elementary school	4 (1.1)
Primary school completed	11 (2.9)
Secondary school completed	80 (21.4)
Higher technical education	137 (36.6)
University	104 (27.8)
Postgraduate degree	38 (10.2)
Monthly family income (USD)^d^	388 (258–789)
Parents vaccinated with COVID-19 vaccine	363 (97.1)
Participation (of the parent) in a clinical trial on COVID-19	22 (5.9)
Parent work in health area	71 (18.9)
Male children	199 (53.2)
Children’s age (years)^d^	14(12–15)
Children with comorbidity^f^	41 (11.0)
Children with any active health insurance	328 (87.7)
Children up to date with the immunization program excluding non-COVID-19 vaccinations (by self-report)	331 (88.5)
Children already vaccinated against COVID-19 (by self-report)	206 (55.1)
Acceptance of COVID-19 immunization in unvaccinated child (*n* = 168)	
Yes	156(41.7)
No	12 (3.2)
Did SARS-CoV-2 infection of you or a close family member/friend had a significant emotional impact on you?^g^	77 (24.7)
Did SARS-CoV-2 infection of you or a close family member/friend had a significant emotional impact on you?^h^	41 (23.2)
Did SARS-CoV-2 infection of you or a close family member/friend had a significant emotional impact on you?^i^	114 (47.7)

^a^Ancón, Ate, Carabayllo, Chaclacayo, Cieneguilla, Comas, El Agustino Independencia, Los Olivos, Lurigancho-Chosica, Lurín, Pachacamac, Puente Piedra, San Juan de Lurigancho, San Juan de Miraflores, San Martin de Porres, Ventanilla, Villa El Salvador, Villa Maria del Triunfo.

^b^Barranco, Bellavista, Breña, Callao, Cercado de Lima, Chorrillos, Jesús María, La Molina, La Victoria, Lince, Magdalena, Miraflores, Pueblo Libre, Rímac, San Borja, San Isidro, San Luis, San Miguel, San Anita, Surco, Surquillo.

^c^Mean ± standard deviation.

^d^Median (interquartile range).

^e^Includes care or administrative area.

^f^Mostly asthma, thyroid disorders, atopic dermatitis.

^g^Consider *n* = 311. We only included responses from those infected with SARS-CoV-2 or with an infected family member/close friend. Subjects whose emotional impact scores were in the 75th percentile or higher on the Likert scale were considered significantly emotionally affected.

^h^Consider *n* = 177. We only included responses from parents who required oxygen for COVID-19 or had a family member/close friend with oxygen requirement. Subjects whose emotional impact scores were in the 75th percentile or higher on the Likert scale were considered significantly emotionally affected.

^i^Consider *n* = 239. We only included responses from those who had a family member/close friend who died from COVID-19. Subjects whose emotional impact scores are in the were percentile or higher on the Likert scale were considered significantly emotionally affected.

### Knowledge and practices about COVID-19 vaccination

Regarding parents’ knowledge about COVID-19 vaccination, we found that about a half (52.1%) had the correct answer about the WHO-approved vaccine (BNT162b2, developed by Pfizer-BioNTech) for children aged 12–17 years. Also, many parents (58.0%) answered correctly that COVID-19 vaccination prevent death or severe illness and were able to adequately recognize solicited adverse events after a COVID-19 vaccination. In contrast, only 20.6% had a correct answer about the underlying approved vaccine mechanism (messenger RNA). About parents’ practices recommended for preventing COVID-19, most reported using face masks (93.3%) and hand sanitizing (79.1%). Interestingly, responses varied by variant epidemiological wave (Supplementary Material 3). Additionally, most parents believed that COVID-19 vaccine studies fulfilled high quality standards. Half of the responders also reported that the brand is crucial for vaccine acceptance (Supplementary Material 4).

### Associated factors with study outcomes

The bivariate analysis included sociodemographic factors and COVID-19 vaccination characteristics. Results with a *p*-value<0.05 in the bivariate regression (Supplementary Materials 5–7) were included in the regression model.

### Children’s participation in a COVID-19 vaccine clinical trial

Approximately 36.6% (137/374) of parents would accept the participation of their children in a COVID-19 vaccine clinical trial. In the multivariate analysis we found that parental age (adjusted PR 0.97; 95% CI 0.96–0.99; *p* = 0.003), and parents with children already vaccinated (adjusted PR 0.67; 95% CI 0.51–0.87; *p* = 0.003) had a lower willingness to enroll their children in a COVID-19 vaccine clinical trial. Conversely, we found a higher willingness of clinical trial enrolment in parents that had participated in a clinical trial of a COVID-19 vaccine (adjusted PR 2.12; 95% CI 1.58–2.86; *p* < 0.01), in parents who believed that COVID-19 vaccines met high-quality standards (adjusted PR 4.49; 95% CI 1.25–16.06; *p* = 0.02), worked in the health area (adjusted PR 1.54; 95% CI 1.16–2.05; *p* = 0.003) or were emotionally affected by SARS-CoV-2 infection (adjusted PR 1.57; 95% CI 1.19–2.06; *p* = 0.003) (Table 2).

**Table 2:** Factors influencing parental willingness to enroll their children aged 12–17 in a potential COVID-19 vaccine trial in Lima, Perú Nov 2021 – Apr 2022.

Factors	Total *n* = 374	Willingness to participat in a COVID-19 clinical trial		Adjusted PR (95% CI)	*p*-value
		No (*n* = 237)	Yes (*n* = 137)		
Do you consider that the COVID-19 vaccine studies meet quality standards?					
No	22	19 (86.4)	3 (13.6)	Ref.	–
Neither agree nor disagree	67	54 (80.6)	13 (19.4)	2.58 (0.66–10.13)	0.18
Yes	285	164 (57.5)	121 (42.5)	4.49 (1.25–16.06)	0.02
Did the SARS-CoV-2 infection on you or a family member/close friend emotionally affect you significantly?^b^					
No	234	156 (66.7)	78 (33.3)	Ref.	–
Yes	77	39 (50.7)	38 (49.3)	1.57 (1.19–2.06)	0.001
Is your child up to date with his/her non-COVID-19 immunizations?					
No	43	35 (81.4)	8 (18.6)	Ref.	–
Yes	331	202 (61.0)	129 (39.0)	1.56(0.89–2.76)	0.12
Is your child vaccinated against COVID-19?					
No	168	96 (57.1)	72 (42.9)	Ref.	–
Yes	206	141 (68.5)	65 (31.6)	0.67 (0.51–0.87)	0.003
Age of parent					
–				0.97 (0.96–0.99)	0.003
Parent participating in any clinical trial on COVID-19 vaccine					
No	352	234 (66.5)	118 (33.5)	Ref.	–
Yes	22	3 (13.6)	19 (86.4)	2.12 (1.58–2.86)	<0.001
Parent working in health care?^a^					
No	303	203 (67.0)	100 (33.0)	Ref.	–
Yes	71	34 (47.9)	37 (52.1)	1.54 (1.16–2.05)	0.003

*Adjusted for the factors included in the table.

^a^Includes health care workers and administrative area.

^b^Consider *n* = 311. We only included responses from those infected with SARS-CoV-2 or with an infected family member/close friend. Significant involvement was greater than the 75th percentile.

PR, prevalence ratio; 95% CI, 95% confidence interval. Ref, Reference, stratum for comparison of effect.

### Interchangeability of COVID-19 vaccines

Approximately a third of the respondent parents (124/374) will accept different COVID-19 vaccine brands between the first and second dose. We found a higher prevalence of acceptance for interchangeability among parents who believed that COVID-19 vaccines clinical trials met quality standards (adjusted PR 10.02; 95% CI 1.40–71.95; *p* = 0.02). Parents who responded during SARS-CoV-2 Delta variant predominance (adjusted PR 0.74; 95% CI 0.54–0.99; *p* = 0.049) and those who consider the vaccine brand is decisive to accept vaccination (adjusted PR 0.44; 95CI %0.31–0.62; *p* < 0.001) were more reluctant to use different vaccines. (Table 3).

**Table 3:** Factors influencing parental acceptance to administer different COVID-19 vaccine brands in adolescents aged 12–17 years in Lima-Peru Nov 2021 – Apr 2022.

Factors	Total *n* = 374	Vaccine interchangeability		Adjusted PR* (95% CI)	*p*-value
		No (*n* = 250)	Yes (*n* = 124)		
Do you consider that the COVID-19 vaccine studies meet quality standards?					
No	22	21 (95.5)	1 (4.5)	Ref.	–
Neither agree nor disagree	67	54 (80.6)	13 (19.4)	4.93 (0.65–37.20)	0.12
Yes	285	175 (61.4)	110 (38.6)	10.02(1.40–71.95)	0.02
Do you consider that the brand of the vaccine is decisive to accept the vaccination?					
No	78	41 (52.6)	37 (47.4)	Ref.	–
Neither agree nor disagree	101	56 (55.5)	45 (44.5)	0.95 (0.71–1.28)	0.74
Yes	195	153 (78.5)	42 (21.5)	0.44 (0.31–0.62)	<0.001
Predominant variant by epidemiologic wave^a^					
Omicron variant predominance	225	140 (62.2)	85 (37.8)	Ref.	–
Delta variant predominance	149	110 (73.8)	39 (26.2)	0.74 (0.54–0.99)	0.049

*Adjusted for the factors included in the table.

^a^In Peru, from November and December 2021 the Delta variant predominated, and from January to April 2022 it coincides with the third COVID-19 wave (predominance of Omicron).

PR, Prevalence ratio; 95% CI, 95% confidence interval. Ref, Reference, stratum to compare the effect.

### Willingness to accept the third dose of COVID-19 vaccine

Most of the parents 90.4% (338/374) reported that they would give their children a third booster vaccine dose. In the multivariate analysis, parental belief that COVID-19 vaccines studies meet quality standards was associated with a higher third dose acceptance (adjusted PR 3.25; 95% CI 1.57–6.74; *p* = 0.002) (Table 4).

**Table 4:** Influencing factors for parental acceptance of a third COVID-19 vaccine dose in their children aged 12–17 years in Lima, Perú Nov 2021 – Apr 2022.

Factors	Total *n* = 374	Acceptance of third COVID-19 vaccine dose		Adjusted PR (95% CI)	*p*-value
		No (*n* = 36)	Yes (*n* = 338)		
Do you consider that the COVID-19 vaccine studies meet quality standards?					
No	22	16 (72.7)	6 (27.3)	Ref.	–
Neither agree nor disagree	67	9 (13.4)	58 (86.6)	2.97 (1.44–6.12)	0.003
Yes	285	11 (3.9)	274 (96.1)	3.25 (1.57–6.74)	0.002
Is your child vaccinated against COVID-19?					
No	168	23 (13.7)	145 (86.3)	Ref.	–
Yes	206	13 (6.3)	193 (93.7)	1.04 (0.98–1.1)	0.18
Does your child have any active health insurance?					
No	46	10 (21.7)	36 (78.3)	Ref.	–
Yes	328	26 (7.9)	302 (92.1)	1.06 (0.95–1.17)	0.30
Parent vaccinated against COVID-19?					
No	11	7 (63.6)	4 (36.4)	Ref.	–
Yes	363	29 (8.0)	334 (92.0)	1.2 (0.66–2.18)	0.55

*Adjusted for the factors included on the table.

PR, Prevalence ratio; 95% IC, 95% confidence interval. Ref, Reference, stratum to compare the effect.

## Discussion

Our study has found that most parents would apply a third COVID-19 vaccine dose to their children and approximately a third would accept the vaccination of their children with different vaccine brands and enroll their children in a COVID-19 vaccine trial. Believing COVID-19 vaccines studies fulfilled high quality standards was associated with the three study outcomes.

Despite lockdown strategies, children contributed significantly to the spread of SARS-CoV-2, especially during the Omicron wave (16, 19). Vaccines for adolescents have been available in Perú since November 2021 (4). However, parental hesitancy regarding their children’s vaccination has been an obstacle for achieving a good vaccination coverage for the first dose series (20, 21) as well as for a third vaccine dose. Our study shows that 90.4% of the parents were willing to give their children a third COVID-19 vaccine dose. This acceptance is very high compared to other studies (around 60%) and even higher compared to adults (22, 23). Also, we found that parents who were more likely to accept the third dose for their children believed that COVID-19 vaccine studies met high quality standards. Easy access to information and high parental level of education in our sample could explain this high prevalence (24, 25). Supporting our findings, a study performed in ten low-middle income countries from 3 different continents, reported that vaccination acceptance increases in hypothetical scenarios of high efficacy and safety profiles (26).

In relation to children’s participation in a COVID-19 vaccine clinical trial, 38.7% parents would accept it. This lower acceptance may be due to lack of experience with the vaccine in pediatric population given the recent introduction in Perú. Parents may have concerns about vaccine safety in children (27). In contrast, adults in Perú (44.1%) (27) and in France (47.6%) (28) had a slightly higher acceptance rate. In addition, the high mortality risk observed in Peru with the Delta and Lambda variants may have influence adults and parents to see a vaccine trial as a way to have early access to an effective vaccine (29, 30).

Regarding vaccine interchangeability, only 33.2% of parents would accept different vaccine brands between the first and second doses. A Saudi-Arabian study found that almost half of the participants would only accept a different vaccine brand if enough information about its safety was given. Also, nearly 60% of participants will only receive different doses if its mandatory (31). According to a qualitative study performed in 2021, where a group of 30 participants were interviewed about COVID-19 vaccine hesitancy, people in Perú have a greater perceived trust in vaccines that are produced in high income countries like the United States and United Kingdom (32). This characteristic correlates with our study’s results about higher interchangeability acceptance among parents with confidence in vaccine quality standards.

Our study has several limitations. Data was collected from November 2021 to April 2022, knowledges, practices and opinions may have changed since parents responded to this questionnaire. The study was only done in Metropolitan Lima, with significant economic diversity, where social media access is limited in low-income settings, introducing a selection bias toward more educated, better informed, tech-savvy subjects that may have responded our on-line questionnaire. Our cross-sectional design will only capture the opinion on the three study outcomes at the survey time. It is known that those perceptions are not static and may change over time, an important variation not captured by our study. Finally, because this is an exploratory study, our findings cannot be generalized to the whole Metropolitan Lima (not a representative sample), or even Perú, meaning that conclusions should be taken with caution. Also, since this was an online self-report questionnaire, some participants may not have accurately remembered specific information for certain questions, introducing a recall bias. However, our study is unique showing parental acceptance of a third vaccine dose, vaccine brand interchangeability and potential participation of children 12–17 years in a vaccine trial in Peru, a country classified as lower- and middle-income level by the World Bank, where this type of information is important for pandemic prevention and control (33). We have not identified similar studies published in the literature. The advertisement in popular social media networks with an on-line questionnaire minimized the risk of selection bias as would have been present in surveys conducted in health facilities or vaccination centers.

In conclusion, most parents would accept a third vaccine dose and approximately a third would allow their children participation in a COVID-19 clinical trial and the use of different vaccine brands in more than one dose. The perception that COVID-19 vaccines trials fulfilled high quality standards was associated with the study outcomes. Our data suggest that as part of the control and prevention of a new pandemic threat, where new vaccines may be use, campaigns focused on high quality standards for vaccine approval and vaccine safety should be conducted to diminish vaccine hesitance in general, and in children in particular. The success of such campaigns should be evaluated by similar surveys conducted in key regions of affected countries and repeated over time to monitor perceptions over time and how they are related to immunization coverage.

## Data Availability

The raw data supporting the conclusions of this article will be made available by the authors, without undue reservation.
