# Supplementary material for: Acceptance of a third COVID-19 vaccine dose, vaccine interchangeability, and clinical trial enrolment among parents of children 12–17 years in Lima, Perú
Source: Front Public Health. 2024 Aug 14;12:1421746. doi: 10.3389/fpubh.2024.1421746 (PMC11349562; doi:10.3389/fpubh.2024.1421746)
Supplement: Supplementary file 1 [file Data_Sheet_1.docx]

Supplementary Material

# **Supplementary Material 1**

**Questionnaire (in Spanish):**

Estimado Sr/Sra:

Gracias por su tiempo, antes de que empecemos con la encuesta, la cual consta de 36 preguntas, es importante que esté informado acerca del estudio, por lo que le pedimos lea atentamente.

Objetivo del estudio: Determinar los conocimientos, actitudes y prácticas de los padres sobre vacunas contra la COVID-19 en adolescentes de 12 a 17 años en Lima-Perú y determinar los factores asociados. Conocer esto es muy importante para implementar estrategias de vacunación en este grupo de edad.

Su participación es completamente voluntaria y no hay ninguna consecuencia si usted no desea participar del estudio. Se respetará la confidencialidad de los datos recolectados y serán usados solo para fines de investigación.

Consentimiento informado:

Yo declaro haber sido informado en una manera clara acerca del estudio y acepto participar en esta investigación. Tengo conocimiento de que mi participación es completamente voluntaria y si los datos de este trabajo son publicados en alguna revista científica serán completamente anónimos.
Habiendo leído el texto:

( ) He comprendido el consentimiento informado y voluntariamente deseo participar en el estudio.
( ) No deseo participar en el estudio.

1. ¿Tiene usted un hijo o hija entre 12 y 17 años?
   1. Sí
   2. No
2. ¿Vacunaría a su hijo entre 12 y 17 años contra COVID-19?
   1. Sí
   2. No
   3. Mi hijo ya está vacunado
3. ¿Aceptaría que su hijo(s) de 12 a 17 años participe en un ensayo clínico de una vacuna experimental contra COVID-19?
   1. Sí
   2. No

Si la respuesta fue No, ¿Podría indicarnos en breves palabras la razón de su decisión?

1. ¿Considera usted que la marca de la vacuna es decisiva para aceptar la vacunación?
   1. Totalmente de acuerdo
   2. De acuerdo
   3. Ni de acuerdo ni en desacuerdo
   4. En desacuerdo
   5. Totalmente en desacuerdo
2. ¿Qué marca de vacuna le colocaría a su(s) hijo(s)?
3. ¿Qué efectos adversos espera después de la aplicación de la vacuna contra COVID-19 en sus hijos de 12 a 17 años? Marque las opciones que considere correctas (puede marcar más de una)
   1. No espero efecto adverso alguno
   2. Dolor en zona de inyección
   3. Fiebre
   4. Convulsiones
   5. Contagio con COVID-19
   6. Malestar general
   7. No sé
4. ¿Qué acciones tomaría si su hijo presenta una reacción leve después de la vacunación contra COVID-19? Marque las que considere correctas. Puede marcar más de uno
   1. Uso paracetamol o similar si tiene fiebre o dolor
   2. Medios físicos (paños fríos, baño con agua tibia) si tiene fiebre calentura
   3. Vigilo, espero que se le pase
   4. Buscar atención médica
   5. Ninguna de las anteriores
5. Si su hijo presenta reacciones en la piel o algún otro efecto adverso leve (dolor en el brazo, dolor de cabeza, fiebre, entre otros) ¿Le colocaría la segunda dosis?
   1. Totalmente de acuerdo
   2. De acuerdo
   3. Ni de acuerdo ni en desacuerdo
   4. En desacuerdo
   5. Totalmente en desacuerdo
6. ¿Le colocaría usted una tercera dosis (de refuerzo) a su hijo?
   1. Sí
   2. No

Si la respuesta fue No, ¿Podría indicarnos en breves palabras la razón de su decisión?

1. ¿Le colocaría usted a su hijo una segunda dosis diferente a la marca de la primera dosis?
   1. Sí
   2. No

Si la respuesta fue No, ¿Podría indicarnos en breves palabras la razón de su decisión?

1. ¿Qué medida(as) de protección usa para protegerse contra la COVID-19? Marque las opciones que considere correctas (Puede marcar más de uno)
   1. Doble mascarilla
   2. Protector facial
   3. Alcohol en gel o lavado de manos
   4. Mi hijo no sale de casa
   5. Ninguna medida
2. ¿Usted mantendría las mismas medidas de prevención contra COVID-19 luego de su vacunación?
   1. Sí
   2. No

Si la respuesta fue No, ¿Podría indicarnos en breves palabras la razón de su decisión?

1. ¿Cree usted que los estudios de vacunas contra COVID-19 cumplen con estándares de calidad?
   1. Totalmente de acuerdo
   2. De acuerdo
   3. Ni de acuerdo ni en desacuerdo
   4. En desacuerdo
   5. Totalmente en desacuerdo
2. ¿En qué formas protege la vacuna contra COVID-19? (Puede marcar más de uno)
   1. Evita el contagio de COVID-19
   2. Evita enfermedad leve por COVID-19
   3. Evita enfermedad moderada por COVID-19
   4. Evita muerte por COVID-19
3. ¿Cuál es la marca de vacuna aprobada por la OMS para adolescentes entre 12 y 17 años?
4. ¿Qué tipo de mecanismo tiene(n) la(s) vacuna (s) contra COVID-19 aprobada(s) en adolescentes entre 12 y 17 años? Marque las opciones que considere correctas (Puede marcar más de uno)
   1. Virus vivo-atenuado
   2. Virus inactivado
   3. ARN mensajero
   4. Vector viral
   5. Proteínas recombinantes
   6. No conozco el mecanismo
5. ¿Sufre alguno de sus hijos alguna enfermedad que requiere atención médica continua?
   1. Sí
   2. No

¿Podría indicarnos la(s) enfermedad(es) que padece?

1. ¿Usted o algún familiar/amigo cercano se ha infectado con COVID-19?
   1. Sí
   2. No
2. Califique y marque del 1 al 10 sobre el grado de afectación que la enfermedad de COVID-19 causó en usted; siendo 0 sin valor alguno y 10 el valor más alto.
   1. 1
   2. 2
   3. 3
   4. 4
   5. 5
   6. 6
   7. 7
   8. 8
   9. 9
   10. 10
3. ¿Usted o algún familiar/amigo cercano ha necesitado utilizar oxígeno debido a la enfermedad COVID-19?
   1. Sí
   2. No
4. Gradúe del 1 al 10 cuánto afectó en usted que algún familiar/amigo cercano haya usado oxígeno debido a COVID-19, siendo 0 sin valor alguno y 10 el valor más alto.
   1. 1
   2. 2
   3. 3
   4. 4
   5. 5
   6. 6
   7. 7
   8. 8
   9. 9
   10. 10
5. ¿Algún familiar/amigo cercano ha fallecido por COVID-19?
   1. Sí
   2. No
6. Gradúe del 1 al 10 cuánto afectó en usted el fallecimiento de esta persona, siendo 0 sin valor alguno y 10 el valor más alto.
   1. 1
   2. 2
   3. 3
   4. 4
   5. 5
   6. 6
   7. 7
   8. 8
   9. 9
   10. 10
7. ¿Participa usted en algún ensayo clínico sobre vacuna contra COVID-19?
   1. Sí
   2. No
8. ¿Usted o su pareja trabajan en el área de salud?
   1. Sí
   2. No
9. ¿Cuentan su(s) hijo(s) menor(es) de 12 a 17 años con algún tipo de seguro de salud activo? (Puede marcar más de uno)
   1. Privado
   2. MINSA
   3. Essalud
   4. Fuerzas armadas
   5. No cuenta con seguro
   6. No sé si cuenta con seguro
10. ¿Su(s) hijo(s) menor(es) de 12 a 17 años están al día con las otras vacunas que le corresponden para su edad (No COVID-19)?
    1. Sí
    2. No
    3. No sabe

Si la respuesta fue No, ¿Podría indicarnos en breves palabras la razón?

1. ¿Usted se ha vacunado contra COVID-19?
   1. Sí
   2. No

Si la respuesta fue No, ¿Podría indicarnos en breves palabras la razón de su decisión?

1. ¿Vive en Lima?
   1. Sí
   2. No
2. ¿En qué distrito de Lima vive usted?
3. ¿Qué edad tiene usted?
4. Indique la relación familiar con su hijo
   1. Padre
   2. Madre
5. De sus hijos entre 12 a 17 años escriba la edad del menor de ellos.
6. Indique el sexo de su hijo
   1. Masculino
   2. Femenino
7. ¿Cuál es su grado de instrucción? (de usted)
   1. Ninguno
   2. Primaria completa
   3. Secundaria completa
   4. Superior técnica
   5. Universitario
   6. Post-grado
8. ¿Cuál es su ingreso económico familiar mensual aproximado en soles? Sume los ingresos de todas las personas que aporten en su hogar y escriba el total (por ejemplo: 950 soles, 1300 soles, 3725 soles, etc)
