# Supplementary figures and images for: Acceptance of a third COVID-19 vaccine dose, vaccine interchangeability, and clinical trial enrolment among parents of children 12–17 years in Lima, Perú
Source: Front Public Health. 2024 Aug 14;12:1421746. doi: 10.3389/fpubh.2024.1421746 (PMC11349562; doi:10.3389/fpubh.2024.1421746)

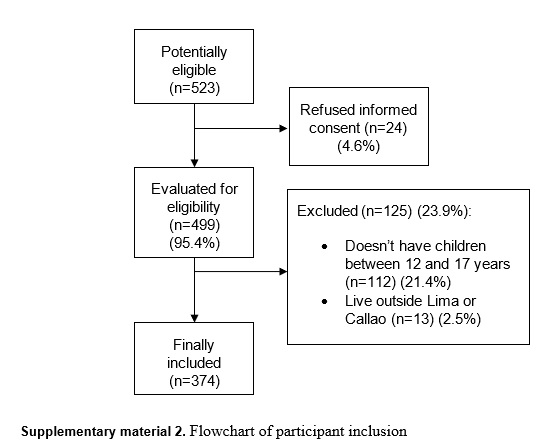

Supplement: Supplementary file 2 [file Data_Sheet_2.docx]

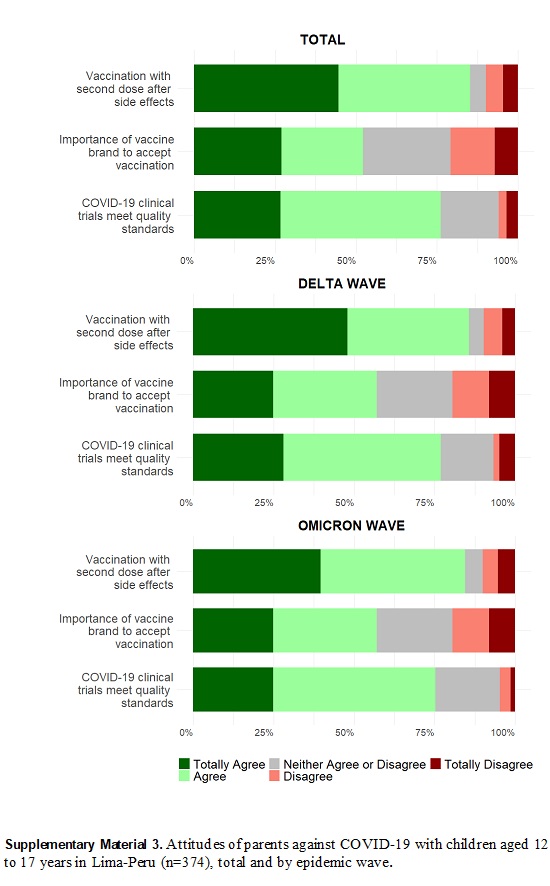

Supplement: Supplementary file 3 [file Data_Sheet_3.docx]
