# Supplementary material for: Acceptance of a third COVID-19 vaccine dose, vaccine interchangeability, and clinical trial enrolment among parents of children 12–17 years in Lima, Perú
Source: Front Public Health. 2024 Aug 14;12:1421746. doi: 10.3389/fpubh.2024.1421746 (PMC11349562; doi:10.3389/fpubh.2024.1421746)
Supplement: Supplementary file 4 [file Data_Sheet_4.docx]

**Supplementary Material 4. Knowledge and practices regarding COVID-19 vaccination of parents with children aged 12 to 17 years in Lima-Peru (n=374).**

| **Characteristics** | **Total  n=374  n (%)** | **Delta variant predominance ^a^  n = 149** | **Omicron variant predominance ^b^ n=225** | **p-value** |
| --- | --- | --- | --- | --- |
| Which is the WHO-approved COVID-19 vaccine for 12-17 years old? | | | | |
| BNT162b2 Vaccine (Pfizer-BioNTech) | 195 (52.1) | 68 (45.6) | 127 (56.5) | 0.02 ^d^ |
| Other vaccines | 163 (43.6) | 70 (47.0) | 93 (41.3) |  |
| Unknown | 16(4.3) | 11 (7.4) | 5 (2.2) |  |
| What is the mechanism of the WHO-approved COVID-19 vaccine 12-17 years? | | | | |
| Messenger RNA | 77 (20.6) | 42 (28.2) | 35 (15.6) | 0.002 ^d^ |
| Other mechanisms | 119 (31.8) | 35 (23.5) | 84 (37.3) |  |
| Unknown | 178 (47.6) | 72 (48.3) | 106 (47.1) |  |
| How does the COVID-19 vaccine protect against COVID-19? ^c^ | | | | |
| Prevents contagion | 64 (17.1) | 27 (18.1) | 37 (16.1) | 0.67 ^d^ |
| Prevents mild disease | 54 (14.4) | 28 (18.8) | 26 (11.6) | 0.05 ^d^ |
| Prevents moderate disease | 74 (19.8) | 24 (16.1) | 50 (22.2) | 0.15 ^d^ |
| Prevents severe disease | 217 (58.0) | 79 (53.0) | 138 (61.3) | 0.11 ^d^ |
| Prevents death | 217 (58.0) | 78 (52.4) | 139 (61.8) | 0.07 ^d^ |
| None | 13 (3,5) | 5 (3.4) | 8 (3.6) | 0.92 ^d^ |
| What are the adverse effects after COVID-19 vaccination? ^c^ | | | | |
| Pain at injection site | 274 (73.3) | 107 (71.8) | 167 (74.2) | 0.61 ^d^ |
| Fever | 143 (38.2) | 50 (33.6) | 93 (41.3) | 0.13 ^d^ |
| General malaise | 142 (37.9) | 47 (31.5) | 95 (42.2) | 0.04 ^d^ |
| Seizures | 9 (2.4) | 3 (2.0) | 6 (2.7) | 1.00 ^e^ |
| Causes SARS-CoV-2 infection | 10 (2.7) | 4 (2.7) | 6 (2.8) | 1.00 ^e^ |
| No adverse effect | 68 (18.2) | 33 (22.2) | 35 (15.6) | 0.11 ^d^ |
| Don't Know | 9 (2.4) | 2 (1.3) | 7 (3.1) | 0.33 ^e^ |
| What action(s) would you take for a mild post-vaccine reaction to COVID-19? ^c^ | | | | |
| Acetaminophen | 343 (91.7) | 131 (87.9) | 212 (94.2) | 0.03 ^d^ |
| Seek medical attention | 37 (9.9) | 12 (8.1) | 25 (11.1) | 0.33 ^d^ |
| Apply physical treatment | 105 (28.1) | 31 (20.8) | 74 (32.9) | 0.01 ^d^ |
| Look out for warning signs | 70 (18.7) | 17 (11.4) | 53 (23.6) | 0.003 ^d^ |
| Wait for it to pass | 68 (18.2) | 16 (10.7) | 52 (23.1) | 0.002 ^d^ |
| None | 6 (1.6) | 0 (0) | 6 (2.7) | 0.09 ^e^ |
| What COVID-19 prevention measure(s) do you use? ^c^ | | | | |
| Use of face mask | 349 (93.3) | 137 (91.95) | 212 (94.22) | 0.39 ^d^ |
| Face shield | 94 (25.1) | 53 (35.6) | 41 (18.2) | <0.001^d^ |
| Alcohol gel or hand washing | 296 (79.1) | 106 (71.1) | 190 (84.4) | 0.002 ^d^ |
| My child does not go out of the house | 89 (23.8) | 33 (22.6) | 56 (24.9) | 0.54 ^d^ |
| None | 4 (1.1) | 1 (0.7) | 3 (1.3) | 1.00 ^e^ |
| Would you maintain the COVID-19 prevention measure(s) post vaccination? | | | | |
| Yes | 371 (99.2) | 148 (99.3) | 223 (99.1) | 1.00 ^e^ |
| No | 3(0.8) | 1(0.7) | 2 (0.9) |  |

1. November and December 2021.
2. January and April 2022.
3. Possibility of checking more than one option, n=374 does not represent 100%.
4. Chi-square test.
5. Fisher's exact test
