# Supplementary material for: Acceptance of a third COVID-19 vaccine dose, vaccine interchangeability, and clinical trial enrolment among parents of children 12–17 years in Lima, Perú
Source: Front Public Health. 2024 Aug 14;12:1421746. doi: 10.3389/fpubh.2024.1421746 (PMC11349562; doi:10.3389/fpubh.2024.1421746)
Supplement: Supplementary file 5 [file Data_Sheet_5.docx]

**Supplementary Material 5. Application of third COVID-19 vaccine dose in adolescents aged 12 to 17 years, in Lima, Perú. Crude regression.**

| **Factors** | **Total n=374** | **Third dose application** | | **Crude PR (95%CI)** | **p-value** |
| --- | --- | --- | --- | --- | --- |
|  |  | **No (n=36)** | **Yes (n=338)** |  |  |
| 1. Do you consider that the COVID-19 vaccine studies meet quality standards? | | | | | |
| No | 22 | 16 (72.7) | 6 (27.3) | Ref. | - |
| Neither agree nor disagree | 67 | 9 (13.4) | 58 (86.6) | 3.17 (1.59 – 6.33) | 0.001 |
| Yes | 285 | 11 (3.9) | 274 (96.1) | 3.53 (1.78 – 6.98) | <0.001 |
| 2. Do you consider that the brand of the vaccine is decisive to accept the vaccination? | | | | | |
| No | 78 | 8 (10.3) | 70 (89.7) | Ref. | - |
| Neither agree nor disagree | 101 | 11 (10.9) | 90 (89.1) | 0.99 (0.89– 1.09) | 0.89 |
| Yes | 195 | 17 (8.7) | 178 (91.3) | 1.02 (0.93 – 1.11) | 0.70 |
| 3. Predominant variant by epidemiologic wave | | | | | |
| Omicron variant predominance | 225 | 18 (8.0) | 207 (92.0) | Ref. | ^-^ |
| Delta variant predominance | 149 | 18 (12.1) | 131 (87.9) | 0.96 (0.89 – 1.03) | 0.21 |
| 4. Is your child vaccinated against COVID-19? | | | | | |
| No | 168 | 23 (13.7) | 145 (86.3) | Ref. |  |
| Yes | 206 | 13 (6.3) | 193 (93.7) | 1.09 (1.01 – 1.16) | 0.02 |
| 5. Is your child suffering from any illness that requires ongoing medical care? | | | | | |
| No | 333 | 30 (9.0) | 303 (91.0) | Ref. | ^-^ |
| Yes | 41 | 6 (14.6) | 35 (85.4) | 0.94 (0.82 – 1.07) | 0.34 |
| 6. Do you (parent) participate in any clinical trial on COVID-19 vaccine? | | | | | |
| No | 352 | 36 (10.2) | 316 (89.8) | - | ^-^ |
| Yes | 22 | 0 (0.0) | 22 (100.0) | - | <0.001^a^ |
| 7. Does either parent work in health care? | | | | | |
| No | 303 | 29 (9.6) | 274 (90.4) | Ref. | - |
| Yes | 71 | 7 (9.9) | 64 (90.1) | 0.99 (0.92 – 1.09) | 0.94 |
| 8. Does your child have any active health insurance? | | | | | |
| No | 46 | 10 (21.7) | 36 (78.3) | Ref. | - |
| Yes | 328 | 26 (7.9) | 302 (92.1) | 1.18 (1.01 – 1.37) | 0.041 |
| 9. Is your child up to date on his/her non-COVID-19 immunizations? | | | | | |
| No | 43 | 5 (11.6) | 38 (88.4) | Ref. | ^-^ |
| Yes | 331 | 31 (9.4) | 300 (90.6) | 1.03 (0.92 – 1.15) | 0.66 |
| 10. Parent vaccinated against COVID-19? | | | | | |
| No | 11 | 7 (63.6) | 4 (36.4) | Ref. | - |
| Yes | 363 | 29 (8.0) | 334 (92.0) | 2.53 (1.16 – 5.54) | 0.02 |
| 11. What is your family relationship with your child? | | | | | |
| Father | 57 | 7 (12.3) | 50 (87.7) | Ref. | ^-^ |
| Mother | 317 | 29 (9.2) | 288 (90.8) | 1.04 (0.93 – 1.15) | 0.51 |
| 12. What is the gender of your child? | | | | | |
| Female | 175 | 19 (10.9) | 156 (89.1) | Ref. | - |
| Male | 199 | 17 (8.5) | 182 (91.5) | 1.03 (0.96 – 1.09) | 0.45 |
| 13. Do you have higher education? ^b^ | | | | | |
| No | 95 | 14 (14.7) | 81 (85.3) | Ref. | - |
| Yes | 279 | 22 (7.9) | 257 (92.1) | 1.08 (0.99 – 1.18) | 0.09 |
| 14. Do you have a monthly family income greater than 780 USD? ^c^ | | | | | |
| No | 264 | 23 (8.7) | 241 (91.3) | Ref. | - |
| Yes | 78 | 5 (6.4) | 73 (93.6) | 1.03 (0.96 – 1.09) | 0.48 |
| 15. Did the SARS-CoV-2 infection of you or a close relative/friend affect you significantly? ^d^ | | | | | |
| No | 234 | 12 (5.3) | 222 (94.9) | Ref. | - |
| Yes | 77 | 10 (13.0) | 67 (87.0) | 0.92 (0.84 – 1.00) | 0.06 |
| 16. Did the need for oxygen use by COVID-19 of you or a close relative/friend affect you significantly? ^e^ | | | | | |
| No | 136 | 11 (8.1) | 125 (91.9) | Ref. | - |
| Yes | 41 | 3 (7.3) | 38 (92.7) | 1.00 (0.91 – 1.11) | 0.87 |
| 17. Did the death by COVID-19 of a family member/close friend affect you in any important way? ^f^ | | | | | |
| No | 125 | 10 (8.0) | 115 (92.0) | Ref. | - |
| Yes | 114 | 9 (7.9) | 105 (92.1) | 1.00 (0.93 – 1.08) | 0.98 |
| 18. Parent's age (years) | | | | | |
| Mean±standard deviation | 42.82± 7.44 | 42.42± 7.33 | 42.86± 7.47 | - | 0.74 ^g^ |
| 19. Child’s age (years) | | | | | |
| Median (RIQ) | 14(12-15) | 14(12-15) | 14(12-16) | - | 0.39 ^h^ |

1. These factors were significantly associated; however, we did not calculate the crude PR, nor were they included in the adjusted model because they contained the value "zero" in one of the cells of the contingency table.
2. Degree of higher education includes technical, university or postgraduate education.
3. Consider n=342, 32 subjects chose not to answer this question. The value of 780 USD corresponds to the 75th percentile.
4. Consider n=311. We only included responses from those infected with SARS-CoV-2 or with an infected family member/close friend. Significant involvement was greater than the 75th percentile.
5. Consider n=177. We only included responses from parents who required oxygen for COVID-19 or had a family member/close friend with oxygen requirement. Significant impairment was greater than the 75th percentile.
6. Consider n=239. We only included responses from those who had a family member/close friend who died from COVID-19. Significant involvement was greater than the 75th percentile.
7. Student's t-test.
8. Mann-Whitney U test.

**PR**: Prevalence ratio; **CI**: Confidence interval. **Ref**: Reference, stratum to compare the effect. **IQR**: Interquartile range.
