# Supplementary material for: Acceptance of a third COVID-19 vaccine dose, vaccine interchangeability, and clinical trial enrolment among parents of children 12–17 years in Lima, Perú
Source: Front Public Health. 2024 Aug 14;12:1421746. doi: 10.3389/fpubh.2024.1421746 (PMC11349562; doi:10.3389/fpubh.2024.1421746)
Supplement: Supplementary file 7 [file Data_Sheet_7.docx]

**Supplementary Material 7. Use of different COVID-19 vaccines in adolescents aged 12 to 17 years, in Lima-Peru. Crude regression.**

| **Factors** | **Total n=374** | **Use of different vaccines** | | **Crude PR (95%CI)** | **p-value** |  |
| --- | --- | --- | --- | --- | --- | --- |
|  |  |  |  |  |  |  |
|  |  | **No (n=250)** | **Yes (n=124)** |  |  |  |
| 1. Do you consider that the COVID-19 vaccine studies meet quality standards? | | | | | |  |
| No | 22 | 21 (95.5) | 1 (4.5) | Ref. | - |  |
| Neither agree nor disagree | 67 | 54 (80.6) | 13 (19.4) | 4.27 (0.59 – 30.88) | 0.15 |  |
|  |  |  |  |  |  |  |
| Yes | 285 | 175 (61.4) | 110 (38.6) | 8.49 (1.24 – 58.09) | 0.03 |  |
| 2. Do you consider that the brand of the vaccine is decisive to accept the vaccination? | | | | | |  |
| No | 78 | 41 (52.6) | 37 (47.4) | Ref. | - |  |
| Neither agree nor disagree | 101 | 56 (55.5) | 45 (44.5) | 0.94 (0.68 – 1.29) | 0.70 |  |
| Yes | 195 | 153 (78.5) | 42 (21.5) | 0.45 (0.32 – 0.65) | <0.001 |  |
| 3. Predominant variant by epidemiologic wave | | | | | |  |
| Omicron variant predominant | 225 | 140 (62.2) | 85 (37.8) | Ref. | ^-^ |  |
| Delta variant predominant | 149 | 110 (73.8) | 39 (26.2) | 0.69 (0.50 – 0.95) | 0.02 |  |
| 4. Is your child vaccinated against COVID-19? | | | | | |  |
| No | 168 | 111 (66.1) | 57 (33.9) | Ref. | - |  |
| Yes | 206 | 139 (67.5) | 67 (32.5) | 0.96 (0.72 – 1.28) | 0.77 |  |
| 5. Is your child suffering from any illness that requires ongoing medical care? | | | | | |  |
| No | 333 | 223 (67.0) | 110 (33.0) | Ref. | ^-^ |  |
| Yes | 41 | 27 (65.8) | 14 (34.2) | 1.03 (0.66 – 1.63) | 0.89 |  |
| 6. Do you (parent) participate in any clinical trial on COVID-19 vaccine? | | | | | |  |
| No | 352 | 239 (67.9) | 113 (32.1) | Ref. | ^-^ |  |
| Yes | 22 | 11 (50.0) | 11 (50.0) | 1.56 (0.99 – 2.43) | 0.05 |  |
| 7. Does either parent work in health care? | | | | | |  |
| No | 303 | 205 (67.7) | 98 (32.3) | Ref. | - |  |
| Yes | 71 | 45 (63.4) | 26 (36.6) | 1.13 (0.80 – 1.60) | 0.48 |  |
| 8. Does your child have any active health insurance? | | | | | |  |
| No | 46 | 32 (69.6) | 14 (30.4) | Ref. | - |  |
| Yes | 328 | 218 (66.5) | 110 (33.5) | 1.10 (0.69 – 1.75) | 0.68 |  |
| 9. Is your child up to date on his/her non-COVID-19 immunizations? | | | | | |  |
| No | 43 | 32 (74.4) | 11 (25.6) | Ref. | ^-^ |  |
| Yes | 331 | 218 (65.9) | 113 (34.1) | 1.33 (0.78 – 2.27) | 0.29 |  |
| 10. Parent vaccinated against COVID-19? | | | | | |  |
| No | 11 | 10 (90.9) | 1 (9.1) | Ref. | - |  |
| Yes | 363 | 240 (66.1) | 123 (33.9) | 3.73 (0.57 – 24.35) | 0.17 |  |
| 11. What is your family relationship with your child? | | | | | |  |
| Father | 57 | 38 (66.7) | 19 (33.3) | Ref. | ^-^ |  |
| Mother | 317 | 212 (66.9) | 105 (33.1) | 0.99 (0.67 – 1.48) | 0.98 |  |
| 12. What is the gender of your child? | | | | | |  |
| Female | 175 | 119 (68.0) | 56 (32.0) | Ref. | - |  |
| Male | 199 | 131 (65.8) | 68 (34.2) | 1.07 (0.79 – 1.42) | 0.66 |  |
| 13. Do you have higher education? ^b^ | | | | | |  |
| No | 95 | 67 (70.5) | 28 (29.5) | Ref. | - |  |
| Yes | 279 | 183 (65.6) | 96 (34.4) | 1.17 (0.82 – 1.66) | 0.39 |  |
| 14. Do you have a monthly family income greater than 780 USD? ^c^ | | | | | |  |
| No | 264 | 180 (68.2) | 84 (31.8) | Ref. | - |  |
| Yes | 78 | 49 (62.82) | 29 (37.18) | 1.17 (0.83 – 1.64) | 0.37 |  |
| 15. Did the SARS-CoV-2 infection of you or a close relative/friend affect you significantly? ^d^ | | | | | |  |
| No | 234 | 150 (64.1) | 84 (35.9) | Ref. | - |  |
| Yes | 77 | 57 (74.0) | 20 (26.0) | 0.72 (0.48 – 1.09) | 0.13 |  |
| 16. Did the need for oxygen use by COVID-19 of you or a close relative/friend affect you significantly? ^e^ | | | | | |  |
| No | 136 | 85 (62.5) | 51 (37.5) | Ref. | - |  |
| Yes | 41 | 30 (73.2) | 11 (26.8) | 0.72 (0.41 – 1.24) | 0.23 |  |
| 17. Did the death by COVID-19 of a family member/close friend affect you in any important way? ^f^ | | | | | |  |
| No | 125 | 87 (69.6) | 38 (30.4) | Ref. | - |  |
| Yes | 114 | 75 (65.8) | 39 (34.2) | 1.13 (0.78 – 1.63) | 0.53 |  |
| 18. Parent's age (years) | | | | | |  |
| Mean±standard deviation | 42.82± 7.44 | 42.86± 7.30 | 42.78± 7.77 | - | 0.86^f^ |  |
| 19. Child’s age (years) | | | | | |  |
| Median (RIQ) | 14(12-15) | 14(12-15) | 14(13-16) | - | 0.08^g^ |  |

1. Higher education level includes technical, university or postgraduate education.
2. Consider n=342, 32 subjects chose not to answer this question. The value of 780 USD corresponds to the 75th percentile.
3. Consider n=311. We only included responses from those infected with SARS-CoV-2 or with an infected family member/close friend. Significant involvement was greater than the 75th percentile.
4. d. Consider n=177. We only included responses from parents who required oxygen for COVID-19 or had a family member/close friend with oxygen requirement. Significant involvement was greater than the 75th percentile.
5. Consider n=239. We only included responses from those who had a family member/close friend who died from COVID-19. Significant involvement was greater than the 75th percentile.
6. Student's t-test.
7. g. Mann-Whitney U test.

**PR**: Prevalence ratio; **CI**: Confidence interval. **Ref**: Reference, stratum to compare the effect. **IQR**: Interquartile range.
